# Impaired LC-NE System—A Novel Molecular Mechanism Underlying Health Disparity and Increased Prevalence of Alzheimer’s Disease Among African Americans

**DOI:** 10.3390/diagnostics16020190

**Published:** 2026-01-07

**Authors:** Yu-Shin Ding, Elizabeth Pirraglia, Jiacheng Wang, Artem Mikheev, Jingyun Chen, Henry Rusinek, James Babb

**Affiliations:** 1 Departments of Radiology, New York University School of Medicine, New York, NY 10016, USAartem.mikheev@nyulangone.org (A.M.); henry.rusinek@nyulangone.org (H.R.); james.babb@nyulangone.org (J.B.); 2 Departments of Psychiatry, New York University School of Medicine, New York, NY 10016, USA; 3 Departments of Population Health, New York University School of Medicine, New York, NY 10016, USA; elizabeth.pirraglia@nyulangone.org

**Keywords:** LC-NE system, PET imaging, health disparity, aging, Alzheimer’s disease, biomarker

## Abstract

**Background:** The current biomarker classification system does not fully explain the increased prevalence of both Alzheimer’s disease (AD) and vascular risk factors for AD—such as diabetes and hypertension--among African Americans (AAs) compared to White participants. Research on cognitive aging has traditionally focused on how declines in cortical and hippocampal regions influence cognition. However, tau pathology emerges decades before amyloid pathology, initially appearing in the brainstem, particularly in the locus coeruleus (LC), the primary source of the brain’s norepinephrine (NE). Further, postmortem studies suggest that the loss of LC neurons is a better predictor of AD symptom severity than amyloid-beta/neurofibrillary tangle pathology in any other brain region. **Methods:** Our decade-long studies in humans using a norepinephrine transporter (NET)-selective radiotracer ([^11^C]MRB) have demonstrated that LC is uniquely vulnerable to aging and stress. In this retrospective study, regression slopes with age (RSAs) for regional NET availability were compared across groups and tested for statistical significance. **Results**: In our primary analysis, higher NET availability was observed in AAs (*N* = 14; 7 males aged 23–49), particularly at younger ages, as compared to White (*N* = 16; 11 males aged 24–55) participants. Our preliminary data also suggest that the rate of decline in NET availability is faster in AAs, with a potential trend toward a more pronounced effect in AA males as compared to White males (e.g., in the left thalamus, RSA was −3.03%/year [95%CI: −5.80% to 1.19%] for AA males vs. RSA = −0.14 for White males [95%CI: −0.79% to 0.47%]. Additionally, in the right anterior cingulate cortex, RSA was −3.4%/year [95%CI: −4.6% to −1.4%] for AA males, compared to RSA = 0.3%/year [95%CI: 0.04% to 1.03%] for White males). **Conclusions:** This report reveals that NET availability (measured with [^11^C]MRB) can serve as a biomarker to index the function of the LC-NE system and that the fast-decline rate of NET in AAs implicates a potential molecular mechanism underlying health disparities observed in the disproportionate AD prevalence.

## 1. Introduction

Despite the racial and ethnic diversity of the global population, significant gaps exist in the scientific literature regarding Alzheimer’s disease (AD) risk factors—including stress, anxiety, sleep disturbances, and vascular disorders—among African Americans (AAs). AAs have been underrepresented in many prominent AD biomarker studies and clinical trials [1]. By 2050, minorities are projected to account for 42% of the nation’s older adults [2], posing critical challenges for aging AAs due to evidence suggesting a two- to three-fold higher prevalence of AD in this group compared to White individuals [2,3,4,5]. The precise reasons for this disparity remain unclear, but limited studies suggest that biological mechanisms may exhibit race-dependent variability, potentially contributing to differences in the manifestation of AD.

Neuroimaging biomarkers in AD have increasingly been used to facilitate earlier diagnoses, enable disease staging, and recruit participants for clinical trials. Positron emission tomography (PET) imaging has played a pivotal role in AD research over the past decade, enabling in vivo accurate detection of Aβ (amyloid-beta) plaques and tau pathology. This has resulted in the well-established ATN framework as follows: amyloid (A), tau (T), and neurodegeneration (N). While a robust connection exists between Aβ and tau, the relationship between Aβ and neurodegeneration remains weak. Current evidence suggests that tau pathology, rather than Aβ, is more strongly associated with brain atrophy, hypometabolism, and cognitive decline [6]. However, racial and ethnic differences in AD biomarkers remain underexplored, and existing data often present conflicting results. For instance, in one of the largest studies to date, performed on the ARIC-PET [7], a community-based cohort without dementia, AAs exhibited a higher burden of amyloid plaques ([^18^F] florbetapir PET imaging) after adjusting for vascular risk factors [7]. Conversely, subsequent study in Washington University cohorts reported no racial differences in brain Aβ burden ([^11^C]PIB) or for Aβ concentration in the cerebrospinal fluid (CSF), while CSF total tau (T-tau) and *p*-tau were lower in AAs than in White participants when both carried *APOE* ε4 gene (*p*  <  0.001) [8]. These results were similar to other studies [9,10], and showed that the degree of cognitive impairment in AAs with lower CSF tau remained comparable to that of White individuals with higher CSF tau burden. This suggests that lower tau levels in AAs do not necessarily offer protection against AD.

Autopsy studies corroborate this complexity. Among confirmed AD cases, AAs and White participants with similar clinical dementia severity demonstrated no significant differences in either Aβ or neurofibrillary tangle (NFT) burdens [11]. Additionally, findings on hippocampal volumetric differences between AAs and Whites have been inconsistent. From the same studies described above, smaller hippocampal volume for AAs than White participants was indicated in one study [8], while other studies reported no racial differences even among cohorts with cognitive normality (CN) or mild cognitive impairment (MCI) [9,10]. However, AAs appear to be more vulnerable to the cognitive effects of cerebrovascular disease than their White counterparts [10].

These findings highlight limitations in the current ATN biomarker classification system (the ATN model [12]: amyloid (A), tau (T), and neurodegeneration (N)), which falls short of explaining the disproportionate prevalence of both AD and vascular risk factors (e.g., hypertension, diabetes) in AAs compared to Whites [3,4,5,13,14,15]. Dr. Williams and others [16,17,18,19,20] have proposed that systemic factors—including residential segregation, discriminatory healthcare practices, and chronic stressors—may influence higher rates of stress [21], anxieties [22], poorer sleep [23,24], and increased prevalence of cigarette smoking, alcohol and substance use, and depression and attention deficit hyperactivity disorders (ADHD) [25], and increased prevalence of both AD and vascular disorders such as hypertension [26,27,28,29,30,31,32], diabetes [33,34], obesity [35], and cardiovascular diseases (CVDs) in AAs [17,22,36,37,38,39], when compared to White participants. Together, they contribute to significantly higher rates for premature mortality [38,40] and AD in this population.

Cognitive aging research has historically focused on cortical and hippocampal decline in relation to cognition, but tau pathology often predates amyloid pathology by decades. Tau pathology initially manifests in the brainstem, particularly in the locus coeruleus (LC), which serves as the brain’s primary source of norepinephrine (NE) [41]. Further, postmortem studies suggest that LC neuron loss is a stronger predictor of the clinical severity of AD symptoms than Aβ or neurofibrillary pathology in cortical or subcortical regions [42,43,44,45,46,47,48]. LC neurons also exert a broad influence over CNS function, particularly in the integration of the adaptive CNS response to various stressors or challenges [49,50], and are a major factor in the pathophysiology of most stress-induced disorders.

Our decades-long research using a norepinephrine transporter (NET)-selective radiotracer ((*S*,*S*)-[^11^C]O-methyl reboxetine; [^11^C]MRB) [51,52] has demonstrated the significant vulnerability of the LC-NE system to numerous conditions, including addiction [53], ADHD [54,55,56], obesity [57,58,59,60,61,62,63], brown fat [64,65], diabetes [66], cardiovascular dysfunction [67], and sleep disorders (review [51,52] and references cited within). Preliminary data on ethnicity- and race-associated differences show that NET availability diminishes more rapidly in AAs compared to Whites. This investigation seeks to elucidate molecular mechanisms underlying health disparities linked to increased AD prevalence and associated vascular risk factors in AAs.

## 2. Materials and Methods

### 2.1. Participants

Healthy adult Black/African American (AA) and non-Hispanic White (nhW, abbreviated as White) participants without current medical conditions and no psychiatric histories were recruited as part of ongoing projects. These participants served as controls for comparison against subjects exhibiting specific disorders (e.g., cocaine addiction, ADHD, obesity, etc.), as described in the Introduction for this retrospective study. All imaging scans were conducted at Yale University using the same MRI and PET scanners with the same acquisition protocol and preprocessing pipeline. All studies employed the same NET-selective radiotracer ([^11^C]MRB), utilizing consistent production and imaging protocols under Dr. Ding’s IND (IND 071928). Each study received approval from the Institutional Review Board (IRB) at either Yale University (the Yale University School of Medicine Human Investigation Committee, HIC) or New York University School of Medicine (NYU Grossman School of Medicine Federal wide Assurance (FWA00004952)). An informed consent was obtained from each participant following a comprehensive explanation of the study. Self-identification served as the basis for race/ethnicity classification.

For this report, data analysis was performed on healthy adults, including AA participants (*N* = 14; 7 M, ages 23–49; mean age = 33.79 ± 7.49) and White participants (*N* = 16; 11 M, ages 24–55; mean age = 39.50 ± 11.04).

### 2.2. Magnetic Resonance Imaging

Magnetic resonance imaging (MRI) was conducted using a 3T Trio (Siemens Medical Systems, Erlangen, Germany) equipped with a circularly polarized head coil. MRI acquisition utilized a sagittal three-dimensional magnetization-prepared rapid gradient-echo sequence (MPRAGE) with the following parameters: echo time (TE) = 3.34 ms, repetition time (TR) = 2500 ms, inversion time (TI) = 1100 ms, flip angle = 7°, and bandwidth = 180 Hz/pixel. Images were acquired at a resolution of 256 × 256 × 176, with a voxel size of 0.98 × 0.98 × 1.0 mm.

### 2.3. PET Imaging and Data Analysis

[^11^C]MRB (a carbon-11 radiolabeled methyl reboxetine) was synthesized using a precursor ((*S*,*S*)-desethyl reboxetine) with high enantiomeric purity (>99.5%), as described previously [68]. Dynamic [^11^C]MRB images were acquired over a duration of 120 min using a High Resolution Research Tomograph (HRRT, CTI/Siemens) scanner and individual structure MRI images were acquired for co-registration purposes.

Post-processing and data analysis on the image data collected from healthy controls (AA and nhW) were conducted at NYU School of Medicine to investigate the potential effects of ethnicity/race on NET availability for this retrospective study. All identifiable info from each participant was erased, and only age, gender, race, and participant code number were used for post-processing and data analysis. Segmentation of cortical and subcortical regions of interest (ROIs) was performed using FreeSurfer (FS2005) based on the MRI atlas templates. Left and right olfactory (L and R Ofac) regions were also generated using the Destrieux Atlas [69]. Subsequent co-registration of PET, MRI, and the FreeSurfer atlas images for each participant was performed using Firevoxel software (developed at NYU, https://firevoxel.org), via mutual information algorithm with autofocus transformation. Time-activity curves (TACs) for each region were generated and [^11^C]MRB uptake in regional NET was quantified as binding potential (BP_ND_). BP_ND_ reflects the density of transporters available for binding [70] and was calculated automatically using the multilinear reference tissue model 2 (MRTM2), with the occipital cortex serving as the reference region (a region with low density of NET and the absence of specific binding, based on our previous studies [55,56]). Further, we have previously shown that MRTM2-derived BP_ND_ estimates highly correlated with BP_ND_ values derived using arterial blood data [56], obviating the need for invasive blood sampling techniques.

The regional NET availability decline rate was then determined via linear regression models of the regional BP_ND_ values regressed on subject ages. The regression slopes with age (RSA, also known as annual percent change (APC) [71]) for each ROI was then estimated based on the linear regression (RSA = 100 × (exp(m) − 1), where m is the estimated slope of the age-decline rate of the regional BP_ND_. The RSA was calculated for all 16 ROIs stratified by race and also by race and sex as four subgroups.

Statistical Analysis: Our primary analysis focused on differences in BP_ND_ by race. The distributions of BP_ND_ by race across all 16 ROIs were first evaluated using exploratory graphical methods. For the 14 bilaterally measured ROIs, values were then averaged across hemispheres to yield 7 ROIs, reducing the number of comparisons and preserving statistical power. Due to the small sample size, the regional differences in BP_ND_ between the two racial groups (classified as African American [AA] vs. White) were assessed using the Wilcoxon rank sum exact test, a nonparametric method that compares the ranks of observations between two independent groups without assuming a normal distribution [72]. The false discovery rate (FDR) was controlled using the Benjamini–Hochberg procedure to adjust for multiple comparisons across the Wilcoxon rank sum exact tests of the bilateral composite ROIs. Both the unadjusted *p*-values and the FDR-adjusted *p*-values (*q*-values) are reported [73].

Differences in age between the two racial groups (classified as African American [AA] vs. White) were assessed using the Wilcoxon rank sum exact test and differences in the distribution of sex across the two racial groups were evaluated using the Fisher exact test [72]. Since there were differences in the age range for AA (age 23 to 49) and White (age 24 to 55) participants, a subset of 12 White participants between the ages of 24 and 49 were selected to match the age range of the 14 AA participants. The mean age of the 12 White participants was 35 (95%CI: 31,39) and for the 14 AA participants it was 34 (95%CI: 30,38).

In sensitivity analyses, an overall composite measure of BP_ND_ was derived by averaging BP_ND_ values across all 16 ROIs for each participant. This composite outcome was intended to provide a global summary of BP_ND_ while reducing regional variability. Linear regression models were then fit with the composite BP_ND_ measure as the dependent variable and race as the primary independent variable, adjusting for age and sex as covariates. These analyses were conducted in both the complete sample (*n* = 30) and a restricted age-matched sample (*n* = 26) to evaluate the robustness of the findings of our primary analysis.

In exploratory analyses, the rate of decline in regional NET availability was determined for each ROI within subgroups defined by sex and race, using linear regression models with regional BP_ND_ values as the dependent variable and age as the predictor. The regression slopes with age (RSAs) for regional BP_ND_ values were then estimated using the following formula: RSA = 100 × (exp(m) − 1), where *m* is the estimated regression slope of age on the regional BP_ND_ values. The bias-corrected and accelerated (BCa) method was employed to derive 95% bootstrap confidence intervals for the RSAs. The subgroups were compared in terms of RSA using a matched-pairs Wilcoxon signed-rank test, as the RSA measurements were considered matched because they were derived from the same ROIs. All statistical tests were conducted at a two-sided 5% significance level using SAS version 9.4 software (SAS Institute, Cary, NC, USA) and IBM SPSS statistics version 28.0.1.1 (14).

## 3. Results

### 3.1. Associations of NET Availability with Race, Sex, and Age

**Figure 1:**
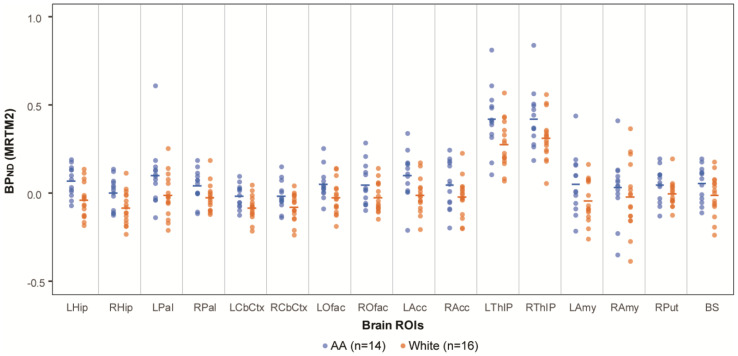
NET availability comparison between AA and White. NET availability (measured as BP_ND_ values of [^11^C]MRB) comparison of AA (*N* = 14; ages 33.8 ± 7.2, blue symbols) vs. White participants (*N* = 16; ages 39.5 ± 10.7; orange symbols). Mean values are shown as a horizontal line for each racial group and ROI.

Across all sixteen investigated brain ROIs, the mean BP_ND_ values (representing NET availability) were consistently higher in AA participants compared to White participants (Figure 1). In the primary analyses, racial differences in BP_ND_ were statistically significant in six of the seven bilaterally averaged ROIs with false discovery rate-adjusted assessment (*q*-value), with AA participants having consistently higher BP_ND_ compared to White participants (Table 1). In the sensitivity analysis of the composite measure of BP_ND_, AA participants had significantly higher BP_ND_ than White participants, both in the model including all participants (*N* = 30, *p* = 0.02) as well as in the age-matched sample (*N* = 26, *p* = 0.02). The covariates of age and sex did not reach significance in either model (all *p* > 0.45).

### 3.2. Race Effects and Sex Effects on Age-Associated Decline Rate of Regional NET Availability

Although neither age nor sex were significantly different between racial groups (Wilcoxon rank sum exact test for age by race, *p* = 0.15; Fisher exact test for sex by race, *p* = 0.46), the absence of statistically significant differences does not imply covariate balance, particularly given the limited sample size. Accordingly, results from subsequent analyses were interpreted as exploratory rather than confirmatory.

**Figure 2:**
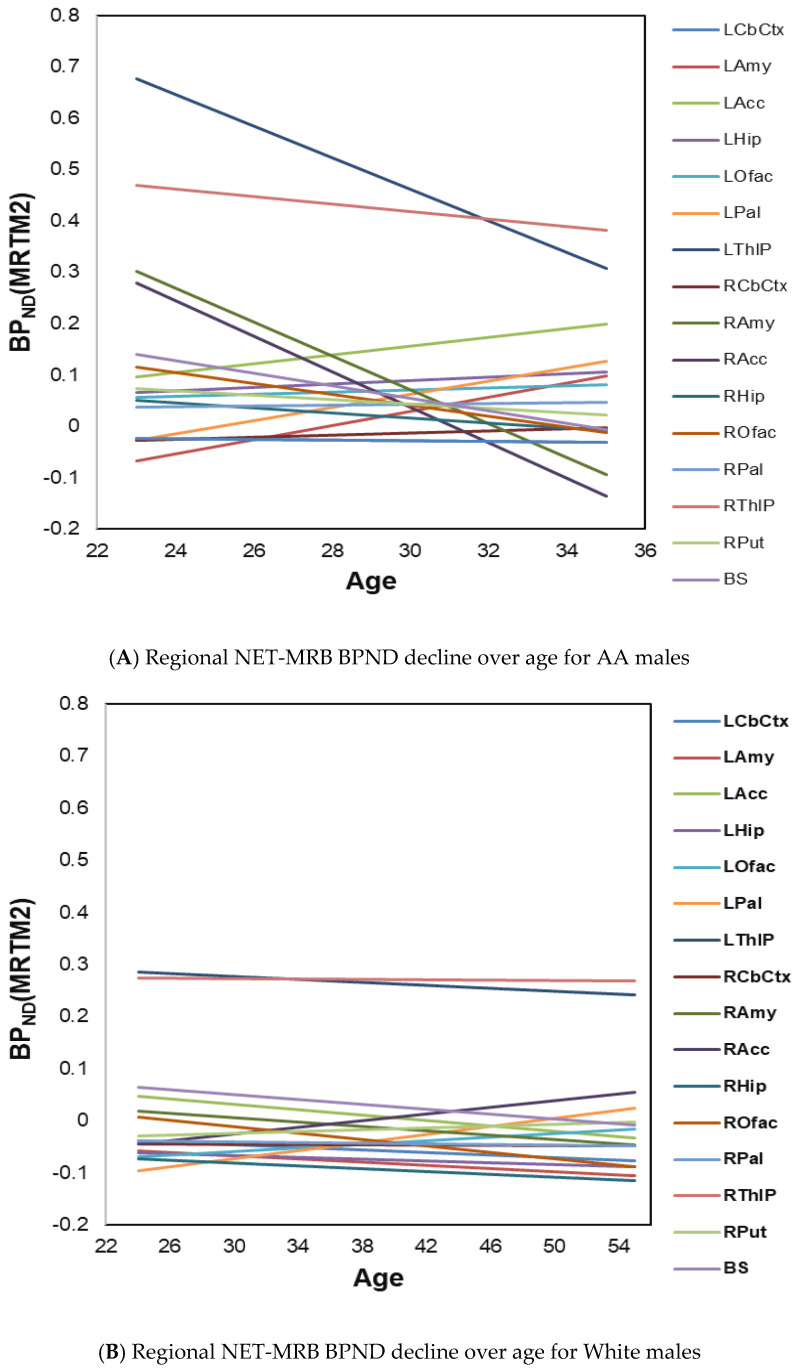
(**A**) Age-effects on regional NET-MRB BP_ND_ decline rates in healthy AA males (*n* = 7, ages 29.7 ± 4.3); (**B**) age-effects on regional NET-MRB BP_ND_ decline rates in White males (*n* = 11, ages 40.4 ± 10.9).

In exploratory analyses, the regression slopes with age (RSAs) in regional BP_ND_ values were then estimated using linear regression models of age predicting NET-MRB BP_ND_ for each ROI within subgroups defined by sex and race. Out of all healthy adults, including both sexes and races (AAs, *N* = 14, ages 33.8 ± 7.2; White, *N* = 16, ages 39.5 ± 10.7), substantially greater age-related decline rates in regional NET-MRB BP_ND_ were observed in AA males (*n* = 7, ages 29.7 ± 4.3). Specifically, AA males showed negative slopes in more brain regions and much steeper declines in some brain regions (Figure 2A) compared to White males (*n* = 11, ages 40.4 ± 10.9) (Figure 2B).

**Figure 3:**
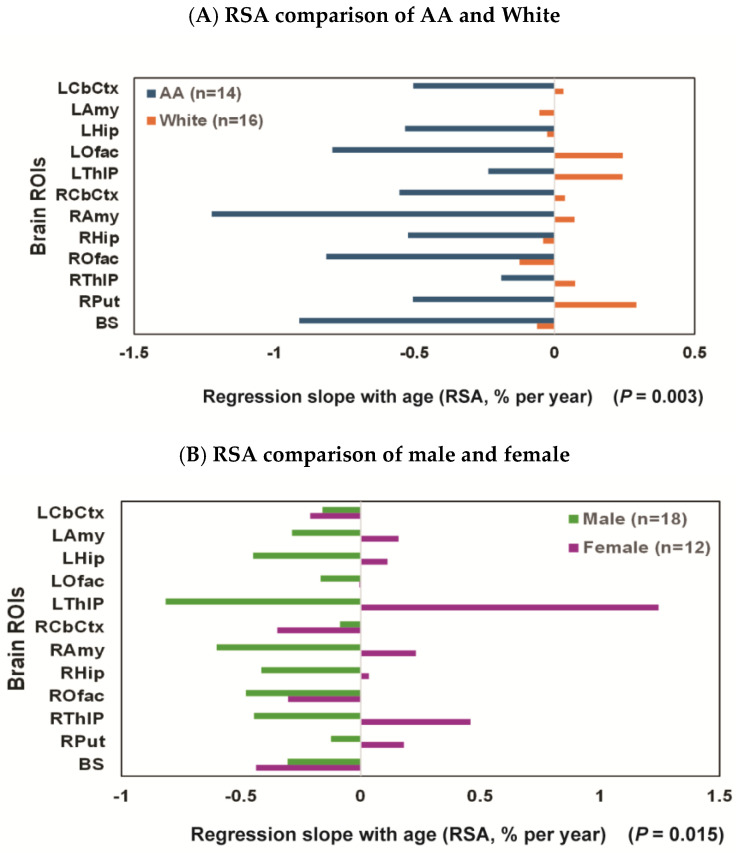

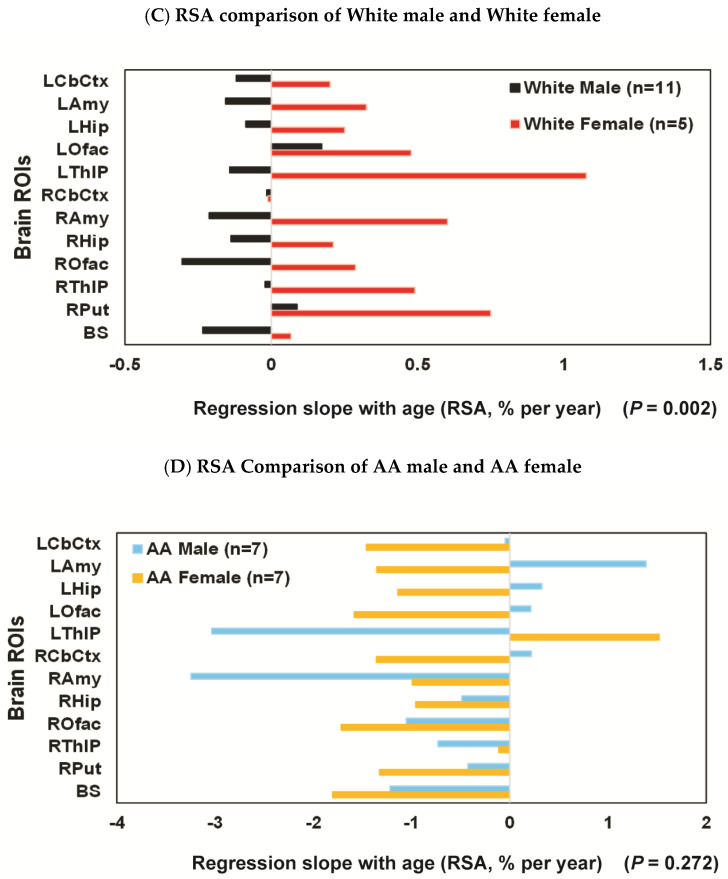
Regression slopes with age (RSAs) comparisons: (**A**) the RSA decline rate for each ROI was consistently faster for AA (*N* = 14; ages 33.8 ± 7.2, blue bars) than for White participants (*N* = 16; ages 39.5 ± 10.7; orange bars); (**B**) female (*N* = 12; ages 25–54 (37.8 ± 9.5); purple bar) vs. male (*N* = 18; ages 23–55 (36.2 ± 9.9); green bar), with the RSA decline rate faster for males than females; (**C**) White female (*n* = 5; ages 37.6 ± 12.4; red bar) vs. White male (*n* = 11; ages 40.4 ± 10.9; black bar), with the RSA decline rate faster for males than females; (**D**) AA female (*n* = 7; ages 37.9 ± 8.0; light orange bar) vs. AA male (*n* = 7; ages 29.7 ± 4.3; light blue bar), the RSA decline rates were high for both AA females and AA males, and the sex difference did not reach significance in this subgroup.

In the exploratory analysis for the effects of race, the decline rates of regional brain ROIs were faster in AAs: e.g., decline rates in BS and ROfac were −0.91% and −0.81%/year, respectively, for AA; by comparison, decline rates in BS and ROfac were −0.06 and −0.13%/year, respectively, for White (Figure 3A). The difference in RSA by race reached significance in the summary measure over 16 ROIs and 12 ROIs both in all participants and in age-matched groups (all *p* ≤ 0.003) (Table 2).

In the exploratory analysis for sex effects [female (*N* = 12, ages 25–54 (37.8 ± 9.5)) and male (*N* = 18, ages 23–55 (36.2 ± 10.2))], there was a significant difference in RSA (***p* = 0.01**) with decline rates faster for males, e.g., −0.8, −0.6, −0.5, −0.4, and −0.4%/year for left thalamus (LThlP), right amygdala (RAmy), right olfactory (ROfac), hippocampus (Lhip & RHip), and right thalamus (RThlP), respectively. While the difference in RSA by sex did not quite reach significance in the summary measure over 16 ROIs (*p* = 0.063) (Table 3), it did reach significance when only 12 ROIs (LCbCtx, Lamy, LHip, LOfac, LThlP, RCbCtx, RAmy, RHip, ROfac, RThlP, RPut, and BS) were included in the computation (***p* = 0.015**) (Figure 3B and Table 3).

The sex effect was also significant within all Whites (*n* = 5, females aged 37.6 ± 12.4 and *n* = 11, males aged 40.4 ± 10.9) with the RSA decline rate faster for males than females both over 16 ROIs (***p* < 0.001**) (Table 3) and 12 ROIs (***p* = 0.002**) (Figure 3C and Table 3). When AA females (*n* = 7; ages 37.9 ± 8.0) and AA males (*n* = 7; ages 29.7 ± 4.3) were compared, the RSA decline rates were high in most regions for both sexes (e.g., −3%/year in LThlP and RAmy and over −1.2%/year in BS and ROfac for AA males vs. −1.8%/year in BS and Ofac for AA females), and the sex difference did not reach significance in these AA subgroups (***p* = 0.272**) (Figure 3D and Table 3). Notably, there appeared a trend suggesting a more pronounced effect in AA males compared to White males (e.g., RSA = −3.03%/year [bootstrap 95%CI: −5.80% to 1.19%] in the left thalamus for AA males vs. RSA = −0.14 for White males [bootstrap 95%CI: −0.79% to 0.47%]. Additionally, in the right anterior cingulate cortex, RSA was −3.4%/year [bootstrap 95%CI: −4.6% to −1.4%] for AA males, compared to RSA = 0.3 cyr [bootstrap 95%CI: 0.04% to 1.03%] for White males).

### 3.3. Representative Images

**Figure 4:**
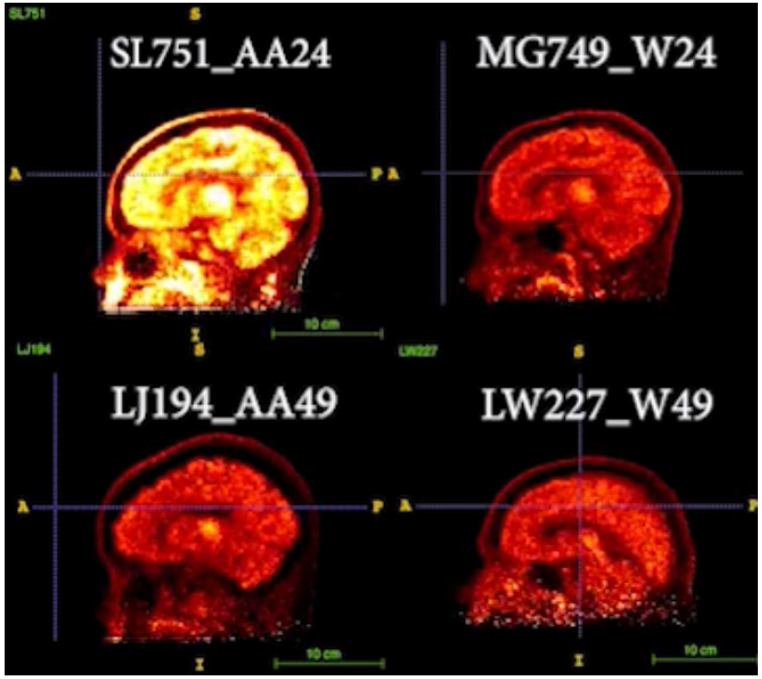
Two pairs of age-matched AA vs. White images are displayed [images of age-24 pair of AA and White (top panel; participant codes of SL751_AA24, MG749_W24, respectively) and age-49 pair of AA and White participants (bottom panel; participant codes of LJ194_AA49, LW227_W49, respectively)]. Their static PET images at the same time frame were compared (occipital was used as the reference region and for setting the contrast). Hot yellow (or bright white) indicated high signal intensity (i.e., higher tracer uptake and binding).

Two pairs of age-matched AA vs. White images were compared in Figure 4 [video images are presented in the Supplemental Section (Video S1) to demonstrate the kinetics of regional tracer uptake over 100 min of the PET dynamic scans after the injection of [^11^C]MRB, starting at *t* = 20 min].

In Figure 4, the static PET BP_ND_ images (occipital as the reference region) at the same time frame after the injection of [^11^C]MRB were compared. As shown in the top panel, the intensity of AA24 is much higher than W24, suggesting higher NET availability of AA than White at younger age (e.g., 24). However, NET density is decreased with age, particularly with a faster decline rate in AA than that in White, as shown in the bottom panel for age 49 participants as follows: the intensities of these images are lower, and the differences between AA and White are not as significant as those displayed in the top panel.

## 4. Discussion

Our current understanding of Alzheimer’s disease (AD) biomarkers and pathological changes derives almost exclusively from research studies involving White participants. For example, the Alzheimer’s Disease Neuroimaging Initiative (ADNI) has provided major insights into the pathological cascade and timeline of AD; however, ADNI-1 included less than 5% African American (AA) participants, limiting its ability to determine whether significant racial differences exist. If AD does exhibit racial disparities, AAs may have increased vulnerability to the disease.

In this relatively young cohort (ages ~20–50), our preliminary results demonstrated that NET availability, measured as binding potential (BP_ND_), is higher in AAs than Whites. This is consistent with research suggesting that stress triggers the release of NE from neurons, leading to increased activity in LC, the brain’s primary source of NE. Chronic stress, a prominent risk factor, has been shown to upregulate NET protein on neuron surfaces, altering how NE is cleared from the synapse and driving dysregulated stress responses [74]. These findings align with the hypothesis that AAs may experience greater chronic stress due to systemic socioeconomic and psychosocial factors.

Furthermore, our results revealed that AAs exhibit significantly faster age-related declines in NET availability compared to Whites, as indicated by regression slopes with age (RSAs) measurements. These findings suggest that PET imaging of NET availability may serve as a potential early biomarker for preclinical AD in midlife—a critical time of biological and psychosocial transition, thus creating a “window of opportunity” for targeting prevention and treatment strategies. This study provides novel insights into the molecular mechanisms potentially underpinning racial and ethnic disparities within the preclinical stages of AD.

In contrast to NET findings, previous imaging studies using [^11^C]PIB have reported no racial differences in brain amyloid burden. Although CSF tau levels showed group differences, current tau radioligands cannot reliably detect early tau pathology in the LC due to their off-target binding with neuromelanin, which is abundant in the LC [75,76,77]. Similarly, recent studies using synaptic vesicle tracers (e.g., [^11^C]UCB­J) to measure synaptic density do not appear sensitive to age effects in most brain regions (the LC remains unexplored) [78]. However, based on our preliminary research across various disorders (e.g., cocaine addiction [53], ADHD [54,55,56,79], obesity [57,58,59,60,61], brown fat [64,65], diabetes [66], cardiovascular dysfunction [67], and sleep disorders; review [51,52] and references cited within), we have demonstrated that (*S*,*S*)-[^11^C]O-methyl reboxetine ([^11^C]MRB) is one of the most effective tracers to date for *in vivo* imaging of LC-NE function in humans. Findings from these studies underscore the broad influence of LC neurons on CNS adaptive responses to stress [49,50], and suggest that LC-NE dysfunction plays a critical role in stress-related disorders [80,81]. This highlights the importance of disrupted LC-NE tone in treating stress-related pathologies and understanding racial health disparities.

Degeneration of the LC has been implicated as a key feature of AD [82,83,84,85], more closely associated with cognitive decline than cell loss in other cortical or subcortical nuclei [86]. Postmortem studies suggest that the loss of LC neurons better predicts the onset and severity of clinical symptoms of AD than Aβ plaques, NFTs, or degeneration in other brain regions [42,43,44,45,46,47]. Our preliminary findings align with social behavioral research on socioeconomic adversity and chronic stress to health disparities in the United States. AAs experience higher rates of chronic stress-related conditions such as hypertension, diabetes, and obesity, all of which are also significant risk factors for AD. Understanding these population-level differences can pave the way for interventions, novel therapeutic targets, and public health strategies aimed at reducing racial/ethnic health disparities, thereby improving the quality of life for aging populations. Continued research should prioritize understanding, preventing, and treating health disparities as a public health imperative.

To conclude, this research provides critical insights into the following: (i) a novel potential marker (NET availability) to detect LC dysfunction associated with increased AD pathology during normal aging; (ii) a potential mechanism linking chronic stress to the disproportionately high prevalence of AD and vascular diseases—including hypertension [26,27,28,29,30,31,32], diabetes [33,34], obesity [35], and cardiovascular conditions—among AAs compared to Whites [17,22,36,37,38,39]; and (iii) the dysfunction of the LC-NE system as a possible driver of health disparities observed in AD expression.

There are some limitations of the study as it did not include information on the measurement of stress levels and other factors that might affect the outcome, such as BMI, socioeconomic status (SES), and education. Additionally, this study did not incorporate our newly adapted imaging methodology using state-of-the-art PET/MR combined scanner with simultaneously acquired and co-registered PET and MR images with a specific neuromelanin-MR sequences, which could provide more precise delineation of the LC region and a more accurate quantification of NET binding. For future studies, we will conduct all these measurements, and include clinical labs, biomarker analysis (e.g., A-beta and tau), and cognitive testing.

Finally, the study is limited by its small sample size, and while our preliminary findings reveal race- and sex-associated effects on NET availability, further studies with larger, systematically matched cohorts are necessary to validate these results. The purpose for publishing these pilot data as a Brief Research Report is to rapidly disseminate our initial findings to the scientific research field and provide a framework for future research to confirm and expand these novel, exploratory but critical observations.

## Figures and Tables

**Table 1:** Analysis of regional NET availability (BP_ND_) with race.

	AA		White		
	Median	IQR ^a^	Median	IQR ^a^	*q*-Value ^b^
Hip	0.06	(−0.04 to 0.12)	−0.07	(−0.13 to −0.02)	0.020 *
Pal	0.09	(0 to 0.15)	−0.04	(−0.08 to 0.04)	0.032 *
CbCtx	−0.01	(−0.08 to 0.05)	−0.08	(−0.13 to −0.04)	0.045 *
Ofac	0.04	(−0.03 to 0.16)	−0.04	(−0.07 to 0.02)	0.038 *
ACC	0.16	(−0.05 to 0.35)	−0.03	(−0.1 to 0.11)	0.034 *
ThlP	0.39	(0.3 to 0.49)	0.28	(0.22 to 0.39)	0.036 *
Amy	0.07	(−0.02 to 0.12)	−0.07	(−0.11 to 0.09)	0.070

^a^ IQR = interquartile range (25th–75th percentile). ^b^ *q*-value = false discovery rate (FDR)-adjusted *p*-value using the Benjamini–Hochberg procedure. * significant difference in BP_ND_ by race with *p*-value or adjusted *p*-value < 0.05.

**Table 2:** Descriptive statistics of RSA by race.

Sample	Regions	Stats	White	AA	Wilcoxon Signed Rank *p* Value *
All participants (*n* = 30)	16 ROIs ^a^	Mean (SD)	0.12 (0.22)	−0.53 (0.45)	<0.001
		Median [IQR]	0.05 [−0.04 to 0.24]	−0.53 [−0.89 to −0.20]	
	12 ROIs ^b^	Mean (SD)	0.06 (0.14)	−0.57 (0.34)	0.003
		Median [IQR]	0.03 [−0.05 to 0.20]	−0.53 [−0.81 to −0.30]	
Age matched ^c^ (*n* = 28)	16 ROIs ^a^	Mean (SD)	0.23 (0.35)	−0.53 (0.45)	<0.001
		Median [IQR]	0.24 [0.02 to 0.29]	−0.53 [−0.89 to −0.20]	
	12 ROIs ^b^	Mean (SD)	0.15 (0.21)	−0.57 (0.34)	0.002
		Median [IQR]	0.22 [0.02 to 0.27]	−0.53 [−0.81 to −0.3]	

^a^ ROIs for comparison (Occi is ref): 16 ROIs (LCbCtx, Lamy, LAcc, LHip, LOfac, LPal, LThlP, RCbCtx, RAmy, RAcc, RHip, ROfac, RPal, RThlP, RPut, and BS) ^b^ Only more relevant 12 ROIs (LCbCtx, Lamy, LHip, LOfac, LThlP, RCbCtx, RAmy, RHip, ROfac, -ThlP, RPut, and BS) were included in the computation. ^c^ A subset of all participants was selected so that the comparison groups were age matched. * *p* values are from the Wilcoxon signed-rank test to compare races and are shown in bold type when indicative of a significant difference (*p* < 0.05).

**Table 3:** Descriptive statistics of RSA by sex.

Sample	Regions	Stats	Male	Female	Wilcoxon Signed Rank *p* Value *
All participants (*n* = 30)	16 ROIs ^a^	Mean (SD)	−0.32 (0.23)	−0.02 (0.50)	0.063
		Median [IQR]	−0.30 [−0.47 to −0.13]	0.01 [−0.34 to 0.22]	
	12 ROIs ^b^	Mean (SD)	−0.36 (0.22)	0.09 (0.45)	**0.015**
		Median [IQR]	−0.36 [−0.47 to −0.16]	0.07 [−0.28 to 0.22]	
White (*n* = 16)	16 ROIs ^a^	Mean (SD)	−0.05 (0.20)	0.47 (0.35)	<**0.001**
		Median [IQR]	−0.10 [−0.20 to 0.06]	0.4 [0.20 to 0.72]	
	12 ROIs ^b^	Mean (SD)	−0.10 (0.14)	0.39 (0.31)	**0.002**
		Median [IQR]	−0.13 [−0.20 to −0.02]	0.31 [0.20 to 0.57]	
AA (*n* = 14)	16 ROIs ^a^	Mean (SD)	−0.58 (1.51)	−0.97 (0.90)	0.234
		Median [IQR]	−0.24 [−1.18 to 0.30]	−1.24 [−1.55 to −0.33]	
	12 ROIs ^b^	Mean (SD)	−0.68 (1.35)	−1.02 (0.92)	0.272
		Median [IQR]	−0.46 [−1.18 to 0.22]	−1.34 [−1.55 to −0.97]	

^a^ ROIs for comparison (Occi is ref): 16 ROIs (LCbCtx, Lamy, LAcc, LHip, LOfac, LPal, LThlP, RCbCtx, RAmy, RAcc, RHip, ROfac, RPal, RThlP, RPut, and BS). ^b^ Only more relevant 12 ROIs (LCbCtx, Lamy, LHip, LOfac, LThlP, RCbCtx, RAmy, RHip, ROfac, ThlP, RPut, and BS) were included in the computation. * *p* values are from the Wilcoxon signed-rank test to compare races and are shown in bold type when indicative of a significant difference (*p* < 0.05).

## Data Availability

The data supporting the findings presented in this study are available within the article. Because the primary study is ongoing, data will not be shared at this time.

## Supplementary Materials

The following supporting information can be downloaded at https://www.mdpi.com/article/10.3390/diagnostics16020190/s1, Video S1: Comparison of Two Pairs of Age-matched AA vs. White MRB-NET video images. Ref. [87] is cited in Supplementary Materials.

## Abbreviations

L (left) or R (right) of CbCtx, Amy, Acc, Hip, Ofac, Pal, ThlP, BS	
CbCtx	cerebellar cortex
Amy	amygdala
Acc	anterior cingulate cortex
Hip	hippocampus
Ofac	olfactory lobe
Pal	pallidum (Globus Pallidus)
THIP	thalamus
BS	brain stem
