# Supplementary material for: Impaired LC-NE System—A Novel Molecular Mechanism Underlying Health Disparity and Increased Prevalence of Alzheimer’s Disease Among African Americans
Source: Diagnostics (Basel). 2026 Jan 7;16(2):190. doi: 10.3390/diagnostics16020190 (PMC12840106; doi:10.3390/diagnostics16020190)
Supplement: Supplementary file 1 [file diagnostics-16-00190-s001.zip › diagnostics-3993678-supplementary.pdf]

Impaired LC-NE System—A Novel Molecular Mechanism Underlying Health Disparity and Increased Prevalence of Alzheimer's Disease Among African Americans

Yu-Shin Ding [1,2,*], Elizabeth Pirraglia [3], Jiacheng Wang [1], Artem Mikheev [1], Jingyun Chen [1], Henry Rusinek [1,2] and James Babb [1]

[1] Departments of Radiology, New York University School of Medicine, New York, NY 10016, USA; artem.mikheev@nyulangone.org (A.M.); henry.rusinek@nyulangone.org (H.R.); james.babb@nyulangone.org (J.B.)

[2] Departments of Psychiatry, New York University School of Medicine, New York, NY 10016, USA

[3] Departments of Population Health, New York University School of Medicine, New York, NY 10016, USA; elizabeth.pirraglia@nyulangone.org

* Correspondence: yu-shin.ding@nyumc.org; Tel.: +1-(212)-263-6605; Fax: +1-(212)-263-7541

Descriptions for the generation of video slides for the PET imaging studies

In the PowerPoint video slides (**Supporting Information, Video S1**), the kinetics of regional tracer uptakes over 100-minutes of the PET dynamic scans, starting at *t* = 20 min after the injection of [^{11}C]MRB, were displayed by using ITKSNAP [1]. ITKSNAP is a powerful software tool designed for interactive image segmentation and visualization in clinical radiology. We first registered the PET to the MR scan of the same subject using a rigid transform with mutual information as the optimization metric via FireVoxel (https://firevoxel.org/). Next, we averaged the dynamic PET image over the frames selected (starting at 20 min, frames 14-33) to generate a new 3D PET image and save as a NIfTI file. After changing the contrast as needed, we exported and saved the files as screenshot series. We used a movie-making program (e.g., ffmpeg) to convert images (e.g., sagittal series to video mp4). To compare two subjects, we changed the contrast maximum in one subject using occipital as the reference region and computed the converting factor. The maximum values of the subject 2 were then be computed. After changing the subject_2's **contrast inspector** maximum value in ITK-SNAP, we saved the individual mp4 video file for subject 2. We then used Adobe Premiere PRO to edit the size of images and made two images display at the same frame and to compare two subjects simultaneously.

Video S1. Comparison of Two Pairs of Age-matched AA vs. White MRB-NET video images (https://www.youtube.com/watch?v=Wy_c87b_HLs)

Two pairs of age-matched AA vs. nhW images were compared in video images to demonstrate the kinetics of regional tracer uptake over 100-minutes of the PET dynamic scans, starting at *t* = 20 min after the injection of [^{11}C]MRB (**Video S1).**

These are dynamic PET-MRB images (occipital was used as the reference region and for setting the contrast) and the movie was prepared with ITK-SNAP. All images display in the same frame with a start time of 20 min. As shown in the top panel, the intensity of AA24 is much higher than W24, suggesting higher NET availability of AA than W at age of 24. NET density is decreased with age, particularly with a faster decline rate in AA than that in W, which is shown in the bottom panel for age 49 participants. The intensities of these images are lower, and the difference between AA and W are not as significant as those displayed in the top panel.

1. Kapur, T., S. Pieper, A. Fedorov, J. C. Fillion-Robin, M. Halle, L. O'Donnell, A. Lasso, T. Ungi, C. Pinter, J. Finet, S. Pujol, J. Jagadeesan, J. Tokuda, I. Norton, R. S. J. Estepar, D. Gering, Hjwl Aerts, M. Jakab, N. Hata, L. Ibanez, D. Blezek, J. Miller, S. Aylward, W. E. L. Grimson, G. Fichtinger, W. M. Wells, W. E. Lorensen, W. Schroeder, and R. Kikinis. "Increasing the Impact of Medical Image Computing Using Community-Based Open-Access Hackathons: The Na-Mic and 3d Slicer Experience." *Med Image Anal* 33 (2016): 176-80.
